# NMCP‐2 polysaccharide purified from *Morchella conica* effectively prevents doxorubicin‐induced cardiotoxicity by decreasing cardiomyocyte apoptosis and myocardial oxidative stress

**DOI:** 10.1002/fsn3.2586

**Published:** 2021-09-15

**Authors:** Na Xu, Yi Lu, Xinmiao Yao, Rui Zhao, Zhebin Li, Jialei Li, Yinglei Zhang, Bo Li, Ye Zhou, Huifang Shen, Liqun Wang, Kaixin Chen, Li Yang, Shuwen Lu

**Affiliations:** ^1^ Institute of Food Processing Heilongjiang Academy of Agricultural Sciences Harbin China; ^2^ Key Laboratory of Zoonosis Research Ministry of Education Institute of Zoonosis College of Veterinary Medicine Jilin University Changchun China

**Keywords:** cardiotoxicity, doxorubicin, mitochondrial apoptosis, NMCP‐2, oxidative stress

## Abstract

Doxorubicin (DOX) is an anthracycline antibiotic used in the clinical treatment of cancer, but its use is limited due to its cardiotoxic effects. Therefore, it is necessary to explore natural compounds that are effective in protecting against the cardiotoxicity caused by DOX. Neutral *Morchella conica* polysaccharides‐2 (NMCP‐2) is a natural polysaccharide with antioxidant activity that was isolated and purified from *Morchella conica* in our laboratory's previous study. This study aimed to investigate the possible protective effect of NMCP‐2 on DOX‐induced cardiotoxicity and the potential underlying mechanisms. The model of DOX‐induced H9C2 cells and the model of DOX‐induced mice were used in this study. In in vitro studies of H9C2 myocardial cells, NMCP‐2 effectively increased the activity of H9C2 cells, reducing the levels of lactate dehydrogenase (LDH). In the mouse model of DOX‐induced chronic cardiotoxicity, NMCP‐2 significantly reduced the cardiac index, reduced the release of serum cardiac enzymes, and improved the pathology of murine myocardial tissues, thereby alleviating DOX‐induced cardiotoxicity. Further mechanism studies showed that pretreatment with NMCP‐2 counteracted the oxidative stress induced by DOX, as indicated by increasing superoxide dismutase (SOD), catalase (CAT), glutathione (GSH) activities, and malondialdehyde (MDA) production decreased. In addition, we observed NMCP‐2 inhibited the activation of the mitochondrial apoptosis pathway and regulated the disordered expression of Bcl‐2 and Bax in the myocardial tissues of DOX‐treated mice. These findings indicated that NMCP‐2, a natural bioactive compound, could potentially be used as a food supplement to reduce the cardiotoxicity caused by DOX.

## Graphical abstract

NMCP‐2 polysaccharide is a natural polysaccharide with antioxidant activity that was isolated and purified from *Morchella conica*. This study aimed to investigate the possible protective effect of NMCP‐2 on DOX‐induced cardiotoxicity and the potential underlying mechanisms. The results showed that NMCP‐2 exerts a definite effect on DOX‐induced myocardial injury. This effect may be associated with the inhibition of oxidative stress and subsequent mitochondria‐dependent apoptotic pathways. Therefore, NMCP‐2 as a natural bioactive compound can be a potential food supplement for reducing the cardiotoxicity caused by DOX.

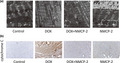

## 1 INTRODUCTION

Doxorubicin (DOX) is a powerful anticancer chemotherapeutic drug, which is widely used in the treatment of cancer clinically (Menna et al., 2012). It is one of the most effective and commonly used broad‐spectrum anticancer drugs for the treatment of malignant tumors (Octavia et al., 2012). However, the clinical application of DOX is severely limited due to its irreversible cardiotoxic effects (Sawyer et al., 2010). Patients treated with DOX even experience severe congestive heart failure many years after stopping DOX chemotherapy, and this condition is difficult to treat with ordinary drugs (Scott et al., 2011). Therefore, many current studies have focused on the development of drugs that can reduce the cardiotoxicity associated with DOX.

DOX‐induced myocardial damage is a complex multifactorial process, mainly related to increased oxidative stress and mitochondrial abnormalities, followed by cardiomyocyte apoptosis (Angsutararux et al., 2015; Minotti et al., 2004). Among these factors, oxidative stress is the key process underlying DOX‐induced myocardial damage. In short, DOX produces large amounts of superoxide anion free radicals and reactive oxygen species (ROS), and then induces mitochondria and cardiomyocyte damage (Damiani et al., 2016; Rochette et al., 2015). Therefore, inhibiting oxidative stress and subsequent apoptosis may be effective treatment methods for counteracting cardiotoxicity caused by DOX. As we all know, the apoptotic pathway is initiated by activation of the p53 tumor suppressor gene. Previous studies have shown that this gene is related to DOX‐induced cardiomyocyte apoptosis (Huang et al., 2011). Proteins of the Bcl‐2 family are divided into two categories: pro‐apoptotic proteins (such as Bax) and anti‐apoptotic proteins (such as Bcl‐2) (Yu & Zhang, 2005). These proteins are key regulators of the mitochondrial‐dependent apoptosis pathway, and p53 can directly regulate the expression of Bcl‐2 family proteins. The activation of p53 changes the balance of pro‐apoptotic proteins and anti‐apoptotic proteins of the Bcl‐2 family, thereby helping to promote cell apoptosis (Brunelle & Letai, 2009). And these proteins can regulate the mitochondrial membrane potential, leading to the release of cytochrome C and the activation of the caspase‐3 enzyme (Crompton, 2000).

Based on these principles, many laboratories have carried out several experimental studies to develop strategies for preventing the cardiotoxic effects of DOX by using various antioxidants and anti‐apoptotic agents. In recent years, more and more natural active substances extracted from animals and plants have been developed into functional foods and natural medicines (Yu, Ji, et al., 2020; Yu, Kan, et al., 2020). At present, it has been found that natural active substances show good prospects for the prevention of cardiotoxicity caused by DOX (Mantawy et al., 2017).

*Morchella conica* is a medical and edible mushroom and has significant medicinal properties and biological activity (Su et al., 2013). Neutral *Morchella conica* polysaccharides‐2 (NMCP‐2) is a polysaccharide purified from *Morchella conica*. Our laboratory's previous study has shown that NMCP‐2 can prevent H_2_O_2_‐induced oxidative stress in the human embryonic kidney (HEK) 293T cells (Xu et al., 2018). However, to the best of our knowledge, no researchers have reported the protective effects and molecular mechanisms of polysaccharide purified from *Morchella conica* against DOX‐induced cardiotoxicity. Therefore, the purpose of this study was to investigate the protective effect of NMCP‐2 on DOX‐induced cardiotoxicity and the potential mechanism.

## 2 MATERIALS AND METHODS

### 2.1 Chemicals and materials

NMCP‐2 was purified from *Morchella conica* in our laboratory (Xu et al., 2018), which was dissolved in 0.9% saline for experiments. DOX (#D1515) and 3‐(4, 5‐Dimethylthiazol‐2‐yl)‐2, 5‐diphenyltetrazolium bromide (MTT) (#M2128) were obtained from Sigma (MO, USA). Bicinchoninic acid protein assay kit (#P0012) and cell lysis buffer kit (#P0013) were obtained from Beyotime (Jiangsu, China). P53 (#2524) was purchased from Cell signaling technology (MA, USA). Cleaved caspase‐3 (ab214430), Cytochrome C (ab133504), Bcl‐2 (ab182858), Bax (ab32503), and β‐actin (ab8226) antibodies were purchased from Abcam (CA, USA), and other chemicals were purchased from Sigma (MO, USA).

### 2.2 Cell culture

H9C2 cells were purchased from the American Type Culture Collection (Manassas, VA, USA) and cultured in Dulbecco's modified Eagle's medium (DMEM) with 10% fetal bovine serum (Hyclone, USA), 100 U/ml penicillin, and 100 mg/ml streptomycin. The cells were maintained at 37℃ in a humid atmosphere under 5% CO_2_ (Xu et al., 2018).

### 2.3 Cell viability

The cell viability was determined using the MTT assay. 5×10^4^ cells/ml H9C2 cells were seeded in 96‐well plates and grown for 24 h. Briefly, the cells in the first group were treated with DOX (1, 2, and 4 μM) for 6, 12, and 24 h. In the second group, the cells were pretreated with NMCP‐2 with different final concentrations (5, 10, 15, and 20 μg/ml) for 12 h. In the third group, 2 μM DOX was added for 24 h at the basis of the second group. Afterward, 10 µl of MTT solution (5 mg/ml) was added and further incubated for 4 h. And then 100 µl of formazan dissolving solution was added and incubated for an additional 4 h. Absorbance at 570 nm wavelength was measured by using a microplate reader (BioTek Instruments, Winooski, VT, USA).

### 2.4 Animals

Female BALB/c mice (6–8 weeks, 18–20 g) were purchased from Liaoning Changsheng Technology Industrial Co., Ltd. (Liaoning, China). All the mice were placed in a suitable environment (24 ± 1℃, light from 6 a.m. to 6 p.m., humidity about 60%). This study was reviewed and approved by the Animal Welfare and Research Ethics Committee at Jilin University.

### 2.5 Animal experimental design

The mice were randomly divided into four groups with ten mice in each group. Group I was served as the control group and received 0.9% saline on 28 days. Group II intraperitoneally (i.p) injection of 0.9% saline (vehicle for DOX) once weekly for 28 days. Groups III received NMCP‐2 50 mg/kg through oral gavage four times per week and i.p injection of DOX (5 mg/kg) once per week. NMCP‐2 was dissolved in 0.9% saline at 25℃ at a concentration of 50 mg/ml. Groups IV received NMCP‐2 only at a dose of 50 mg/kg four times weekly (Mantawy et al., 2017). At the end of 28 days, all the mice were sacrificed under light ether anesthesia. The body weights were measured, and the hearts were quickly harvested, weighed, and processed for the next studies. Blood was collected, and serum was separated for serological studies.

### 2.6 Determination of serum enzyme levels in mice

The levels of serum lactate dehydrogenase (LDH), cardiac injury markers including creatine phosphokinase (CK‐MB), alanine aminotransferase (ALT), and aspartate aminotransferase (AST) activity were measured according to the manufacturer's protocols (Nanjing Jiancheng Institute of Biotechnology, Nanjing, China).

### 2.7 Measurement of malondialdehyde (MDA) level and antioxidant enzyme activities in the cardiac homogenate

The contents of MDA and the activities of antioxidant enzymes, including superoxide dismutase (SOD), catalase (CAT), and glutathione (GSH), were measured in cardiac homogenates according to the manufacturer's protocols (Nanjing Jiancheng Institute of Biotechnology, Nanjing, China).

### 2.8 Histopathological examination

For light microscopy examination, myocardial tissues were fixed in 10% formalin, followed by being embedded in paraffin. 5‐μm‐thick paraffin sections were stained with hematoxylin and eosin (H&E). For the Masson dyeing, the paraffin sections were stained with a Masson's trichrome stain kit (Solarbio, Beijing, China). The slides were examined under a light microscope (Olympus BX‐50).

### 2.9 Electron microscopic examination

The pretreated heart specimens were fixed in 2% osmium tetroxide and dehydrated in graded ethanol. Then, the specimens were infiltrated and embedded by standard techniques. The thin sections (1 µm) were cut and stained with 1% toluidine. Afterward, the ultrathin sections (80 nm thickness) were stained with uranyl acetate and lead citrate and photographed with a transmission electron microscope (Philips CM‐10).

### 2.10 Immunohistochemical examination

3‐μm thickness of paraffin‐embedded tissue sections was rehydrated in xylene and then graded ethanol solutions. The tissue slides were then blocked with 5% bovine serum albumin (BSA) in TBS for 2 h. The sections were then immunostained with one of the following primary antibodies (mouse monoclonal anti‐cytochrome C antibody, rabbit polyclonal anti‐caspase‐3 antibody, or mouse monoclonal anti‐p53 antibody) at a concentration of 1 μg/ml, containing 5% BSA in TBS and incubated overnight at 4℃. Then, the slides were incubated with goat anti‐rabbit secondary antibody. After that, the sections were incubated in 0.02% diaminobenzidine solution containing 0.01% H_2_O_2_ for 5–10 min. Immunohistochemical quantification was analyzed using image analysis software (Image J, 1.46a, NIH, USA).

### 2.11 Western blotting

Myocardial tissues were homogenized in lysis buffer. Then, the protein concentrations were analyzed using a BCA kit. The protein samples were separated by SDS‐polyacrylamide gel electrophoresis and transferred on polyvinylidene difluoride (PVDF) membranes. The membranes were blocked and then incubated with the primary antibodies (Bax, or Bcl‐2) at 4℃. Subsequently, the membranes were incubated with conjugated secondary antibodies at room temperature for 1 h. The band intensities of the blots were quantified using optical densitometry (ImageJ software) (Xu et al., 2018).

### 2.12 Statistical analyses

The data are expressed as the mean ± standard deviation (*SD*). Statistical comparison among the multiple groups was analyzed using a one‐way ANOVA, where *p* < .05 was considered as significant. All the analyzed data were performed using SPSS 20.0 (SPSS, USA).

## 3 RESULTS

### 3.1 NMCP‐2 protected H9C2 cardiomyocytes from DOX‐induced cytotoxicity

**FIGURE 1:**
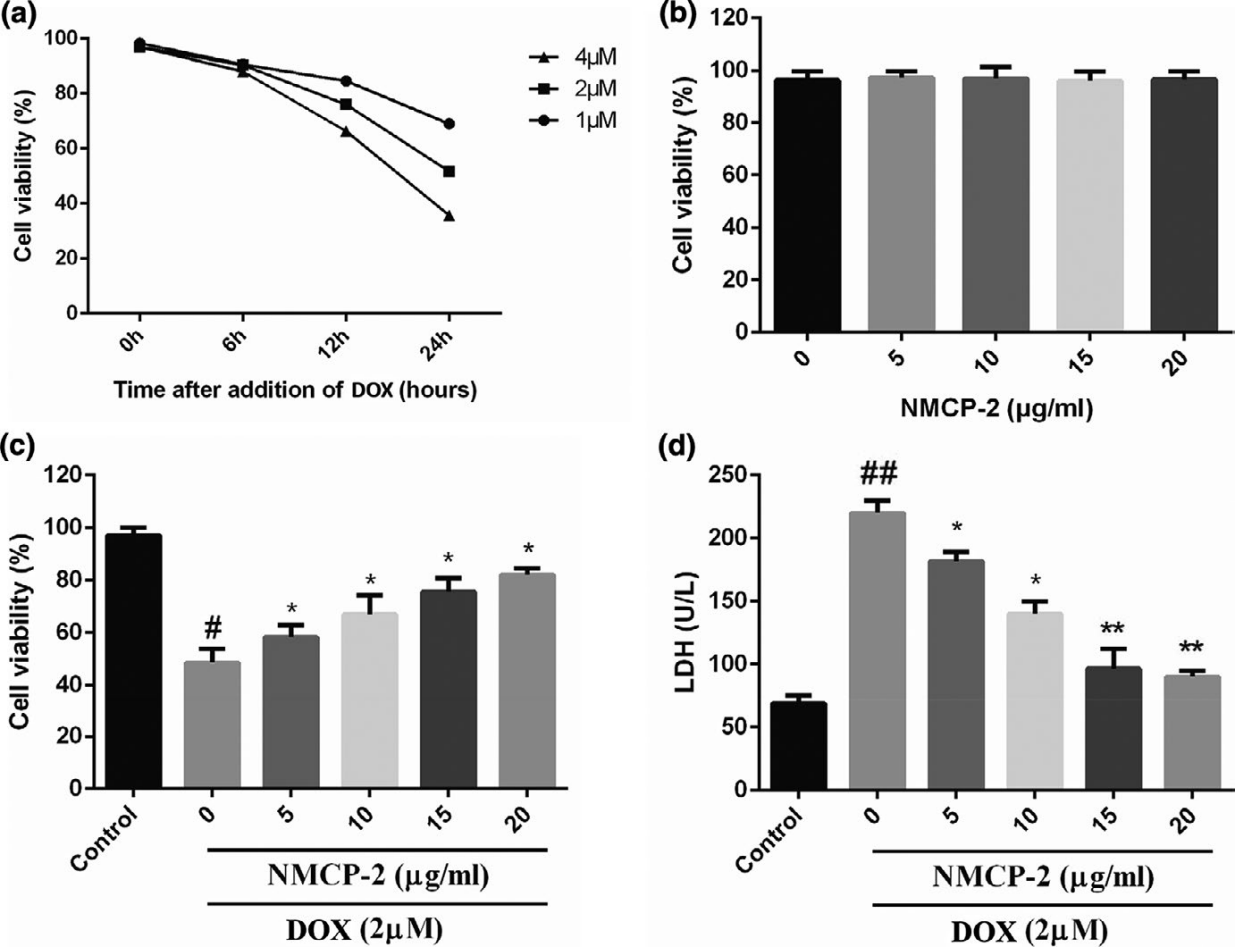
Effects of DOX and NMCP‐2 on cell viability and LDH release in H9C2. (a) H9C2 cardiomyocytes were treated for 6, 12, and 24 h with different concentrations of DOX, and cell viability was determined by MTT assay. (b) H9C2 cardiomyocytes were treated with different concentrations of NMCP‐2 for 12 h, and cell viability was expressed as the relative percentage of the control group. Control and NMCP‐2‐treated cells were further exposed to 2 µM DOX for 24 h. (c) Cell viability. (d) LDH release was measured. Results are represented as means ± *SD* from three independent experiments. ##*p* < .01 and #*p* < .05 versus Control; **p < .01 and *p < .05 versus DOX‐induced group

First, the MTT method was used to determine the effect of DOX on H9C2 cell activity and to evaluate the protective effect of NMCP‐2 on DOX‐induced cardiac injury in vitro. H9C2 cardiomyocytes were treated with different concentrations of DOX (1, 2, and 4 µM) for 6, 12, and 24 h. The results showed that with the increase of DOX dose and treatment time, the cell viability of H9C2 cardiomyocytes continued to decrease (Figure 1a). Approximately 50% of the H9C2 cardiomyocytes died after being treated with 2 μM doxorubicin for 24 h. Therefore, treatment with 2 μM doxorubicin for 24 h was used as the conditions for subsequent in vitro experiments.

The direct effect of NMCP‐2 on H9C2 cardiomyocytes was analyzed before testing the ability of NMCP‐2 protection against DOX‐induced H9C2 cell damage. Cells were treated for 12 h with NMCP‐2 with varying concentrations ranging from 5 µg/ml to 20 µg/ml. As showed in Figure 1b, there was no significant difference in cell viability between NMCP‐2‐treated and control groups. These results indicated that none of the tested NMCP‐2 concentrations can induce H9C2 cardiomyocytes damage.

To investigate the potential protective effects of NMCP‐2 on the DOX‐induced H9C2 cells, H9C2 cardiomyocytes were pretreated with NMCP‐2 (5 µg/ml to 20 µg/ml) for 12 h and then placed in 2 µM DOX for 24 h. Cell viability and the release of LDH were used as criteria for evaluating whether NMCP‐2 exerts protective effects against DOX‐induced cytotoxicity. As shown in Figure 1c,d, with pretreatment of 5, 10, 15, and 20 g/ml NMCP‐2, the cell activity of H9C2 remarkably increased and the release of LDH remarkably decreased. Therefore, NMCP‐2 has the ability to protect H9C2 cardiomyocytes from DOX‐induced cytotoxicity.

### 3.2 NMCP‐2 reduced DOX‐induced cardiotoxicity in mice

**FIGURE 2:**
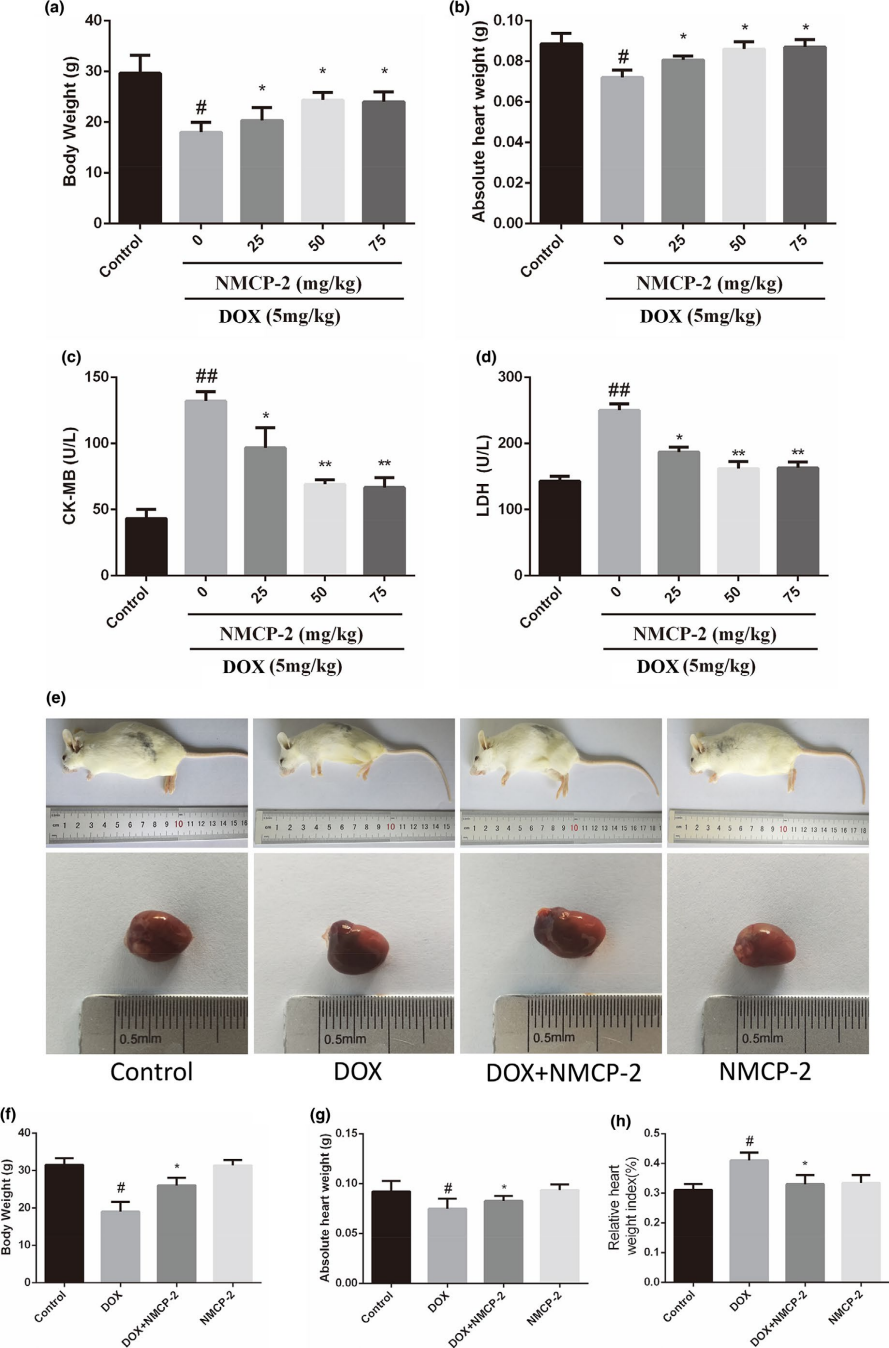
Body and heart weights and cardiotoxicity markers of the mice treated with NMCP‐2 and/or DOX. (a) The body weight of the mice. (b) The heart weight of the mice. (c) CK‐MB activity (U/L). (d) LDH activity (U/L). (e) The appearance of mice and heart (f). The body weight of the mice pretreatment with NMCP‐2 (50 mg/kg). (g) The heart weight of the mice pretreatment with NMCP‐2 (50 mg/kg). (h) The relative heart weight index was measured. Results are represented as means ± *SD* (*n * =  10 per group). ##*p* < .01 and #*p* < .05 versus Control; *p < .05 versus DOX‐induced group

First, the toxicity of DOX and NMCP‐2 was evaluated. As shown in Figure 2a,b, mice were injected intraperitoneally (ip) with DOX (5 mg/kg per dose, cumulative dose of 20 mg/kg over 4 weeks). After 28 days, the body and heart weight of DOX‐induced mice had significantly decreased compared with the control mice. However, in the mice at doses of NMCP‐2 orally (1–75 mg/kg, ip, before DOX administration), the body and heart weight recovered in a dose‐dependent manner. In addition, the expression of serum enzymes CK‐MB and LDH levels in the DOX‐induced group of mice was significantly higher than that in the control group. Compared with the DOX mice, given with 50 mg/kg NMCP‐2 reduced serum CK‐MB and LDH levels by about 50% (Figure 2c,d). A higher concentration of NMCP‐2 (up to 75 mg/kg) showed no additional benefit on the body and heart weights, or CK‐MB and LDH levels. Therefore, a dose of 50 mg/kg was chosen for the subsequent in vivo experiment.

To explore the potential protective effect of NMCP‐2 against DOX‐induced cardiotoxicity in mice, we first evaluated the appearance of the mice in each group. As shown in Figure 2e, the hearts of mice in the DOX‐induced group showed obvious cardiac congestion and swelling, indicating that the heart was seriously injured. Moreover, all the mice in the DOX‐induced group appeared weak and thin. In addition, the heart and body weight of the DOX‐induced group were significantly lower than the control group (*p* < .05). In contrast, the pretreatment with NMCP‐2 (50 mg/kg) in mice resulted in a significant recovery of heart and body weight (Figure 2f,g). On the other hand, the heart weight index of the DOX‐induced group was significantly higher than that in the control group (*p* < .05), and the pretreatment with NMCP‐2 (50 mg/kg) in mice effectively reversed this situation (Figure 2h).

### 3.3 NMCP‐2 ameliorated cardiac fibrosis and dysfunction in mice treated with DOX

**FIGURE 3:**
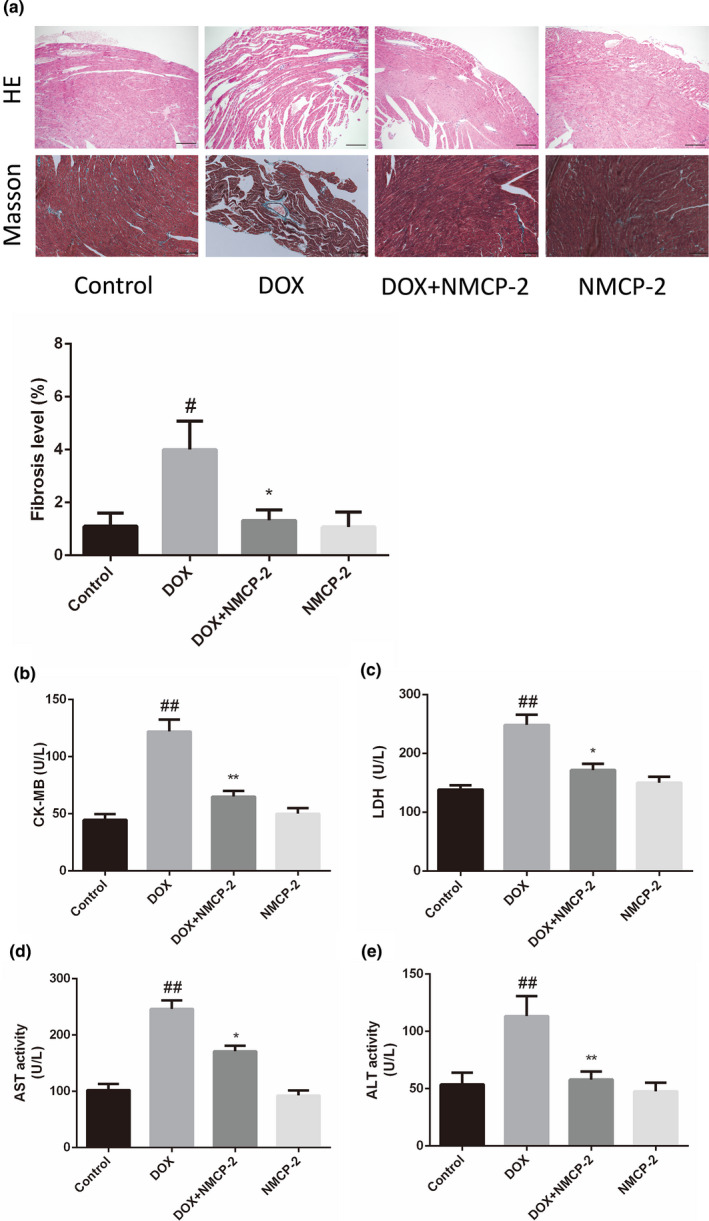
NMCP‐2 alleviated DOX‐induced cardiotoxicity in mice. (a) Effects of NMCP‐2 on HE and Masson staining images of myocardial tissues in mice (scale bar = 100 µm). Quantitative results of cardiac fibrosis were shown below. (b) Effects of NMCP‐2 on serum levels of CK‐MB in mice treated by DOX. (c) LDH activity (U/L). (d) AST activity (U/L). (e) ALT activity (U/L). Results are represented as means ± *SD* (*n * =  10 per group). ##*p* < .01 versus Control; **p < .01 and *p < .05 versus DOX‐induced group

As shown in Figure 3a, HE staining showed that the cardiomyocytes in the control group were arranged neatly with no hemorrhage, edema, and other abnormalities. However, DOX caused myocardial cell damage including obvious myocardial tissue texture unclear, vacuolation, and muscle fibers was broken, which were significantly improved by NMCP‐2. In addition, fibrosis is the main mechanism responsible for DOX‐induced cardiomyocyte dysfunction. By examining the Masson staining under the microscope, an increase in cardiac fibrosis was observed in the hearts of the DOX‐induced mice compared with the control group. The results showed that the structure of the collagen network in the interstitial and perivascular regions was changed and disordered. These results indicated that NMCP‐2 pretreatment significantly reduced pathological changes, and there was no obvious abnormality in the NMCP‐2 group alone.

On the other hand, the activity of some serum markers suggests myocardial injury, including CK‐MB, LDH, AST, and ALT. In Figure 3b‐e, CK‐MB, LDH, AST, and ALT were significantly increased in the DOX‐induced group compared with the control group (*p* < .05). Compared with the DOX group, the CK‐MB, LDH, AST, and ALT activities of mice pretreated with NMCP‐2 were significantly reduced (*p* < .05). However, NMCP‐2 treatment alone did not result in any significant changes in all these markers compared with the control.

### 3.4 NMCP‐2 protected against DOX‐induced oxidative stress and apoptotic damage

**FIGURE 4:**
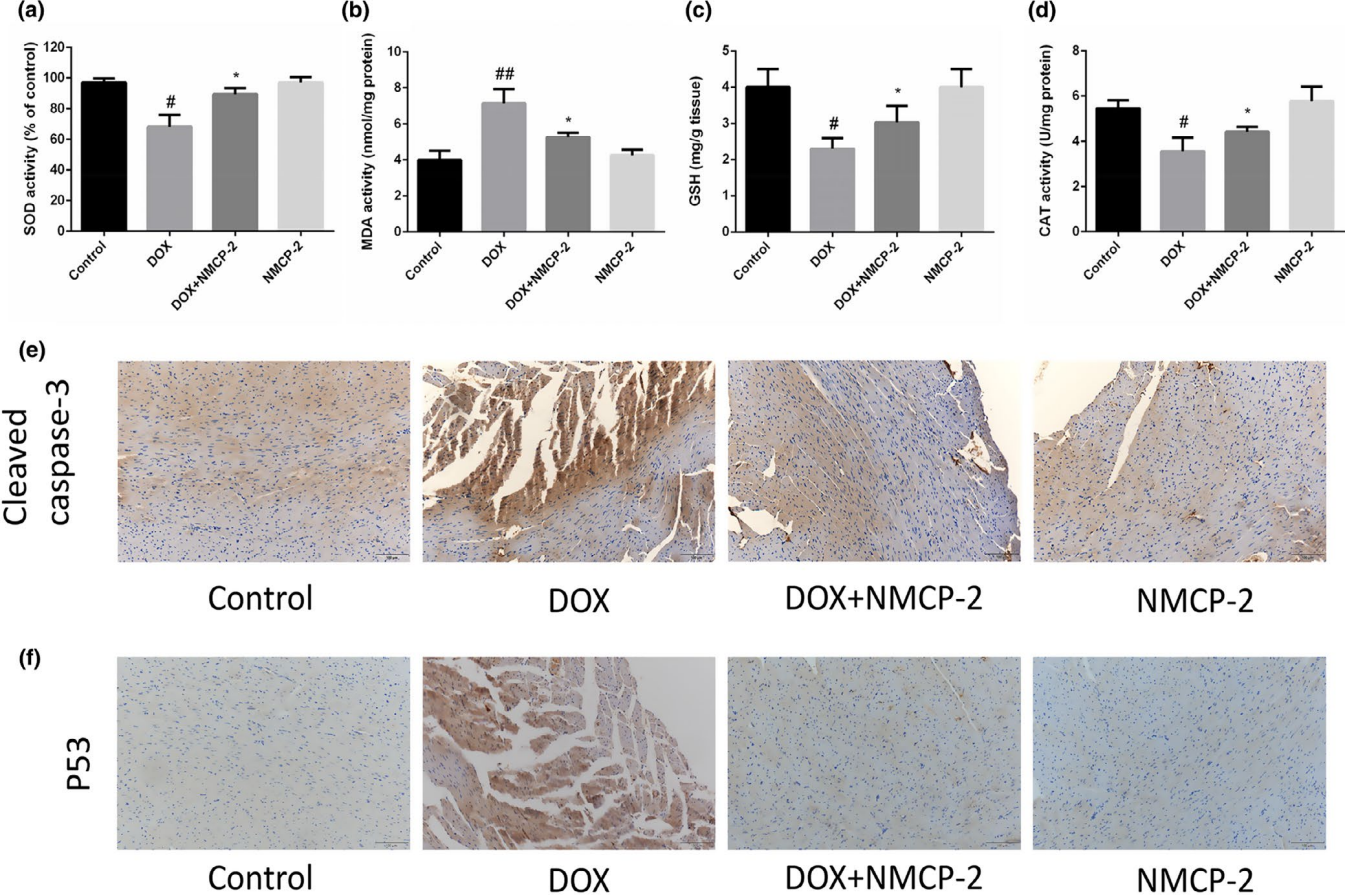
Effects of DOX and NMCP‐2 on oxidative stress and apoptosis in mice. (a) Effects of NMCP‐2 on the levels of SOD in myocardial tissues of the mice treated with DOX. (b) MDA activity (nmol/mg protein). (c) GSH level (mg/g tissue). (d) CAT activity (U/mg protein). (e) Mice heart sections were analyzed by immunohistochemistry with an antibody reactive to cleaved caspase‐3. Scale bar = 100 µm. (f) Mice heart sections were analyzed by immunohistochemistry with an antibody reactive to p53. Scale bar = 100 µm. Results are represented as means ± *SD* (*n * =  10 per group). ##*p* < .01 and #*p* < .05 versus Control; *p < .05 versus DOX‐induced group

Many studies have shown that oxidative stress is the main mechanism underlying DOX‐induced cardiotoxicity (Lin & Yin, 2013; Songbo et al., 2019; Tian et al., 2017; Wojnowski et al., 2005). Thus, we examined the activity of related antioxidant enzymes. As shown in Figure 4a‐d, after DOX treatment, mouse heart‐related antioxidant enzymes such as SOD, CAT, and GSH activity decreased and MDA production increased, but NMCP‐2 effectively improved these changes. These findings indicated that NMCP‐2 treatment decreased the cardiac oxidative stress induced by DOX. To further confirm whether the protective effect of NMCP‐2 against DOX‐induced myocardial damage is related to apoptosis, the protein expression of caspase‐3 and p53 in myocardial tissues was detected by immunohistochemistry (IHC). As shown in Figure 4e,f, the protein expression of caspase‐3 and p53 in myocardial tissues decreased significantly after NMCP‐2 pretreatment. However, NMCP‐2 treatment alone had no significant effect on myocardial cell apoptosis. These results indicated that NMCP‐2 protected against DOX‐induced oxidative stress and apoptotic damage.

### 3.5 The protective effects of NMCP‐2 against DOX‐induced apoptosis were related to the mitochondria‐dependent apoptotic pathway

**FIGURE 5:**
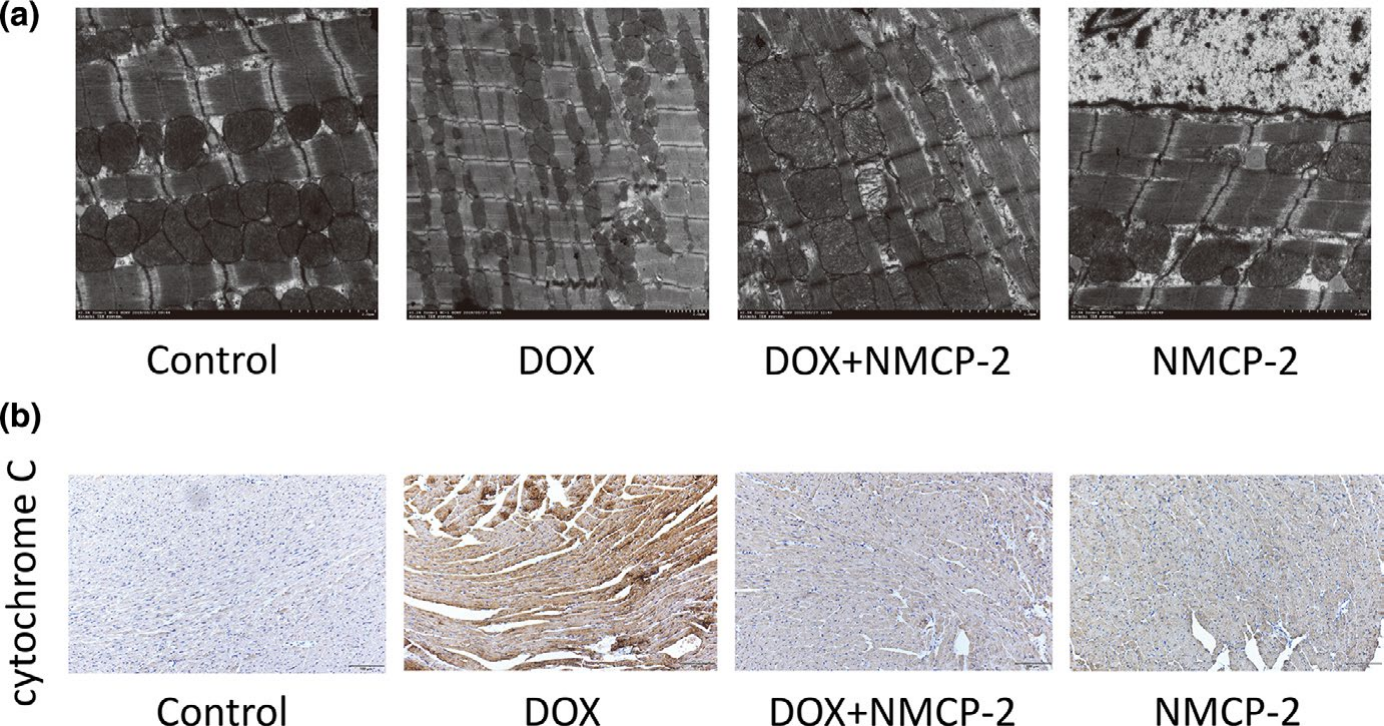
Effects of DOX and NMCP‐2 on the mitochondrial apoptotic pathway in mice. (a) Mitochondrial injuries in myocardial tissues were observed by transmission electron microscopy. Scale bar = 2.0 µm. (b) Protein expression of cytochrome C by immunohistochemical staining (scale bar = 100 µm). Results are represented as means ± *SD* (*n * =  10 per group)

The mitochondria‐dependent apoptotic pathway is considered the main mechanism underlying DOX‐induced cardiomyocyte apoptosis (Shi et al., 2018; Sun et al., 2013). As shown in Figure 5a, transmission electron microscopy showed that DOX‐treated mice exhibited obvious abnormalities in myocardial tissues. Compared with those in the control group, the myocardial tissues induced by DOX have obvious myocardial fiber rupture, disorder of myocardial fiber arrangement, mitochondrial deformation, and obvious cytoplasmic vacuolization. In addition, the mitochondria of the cardiomyocytes in the DOX‐induced group showed swelling, loss of cristae, and disordered arrangement. These structural damages were partly alleviated by pretreatment with NMCP‐2.

Furthermore, mitochondrial cytochrome C release plays an important role in the mitochondria‐dependent apoptotic pathway (Khan et al., 2012). Immunohistochemistry was used to evaluate the expression of cytochrome C protein. DOX induced a significant increase in cytochrome C expression, as shown by the intense brown color. In contrast, the cytochrome C expression was significantly reduced in the mice treated with NMCP‐2 (Figure 5b).

### 3.6 NMCP‐2 modulated the expression of Bcl‐2 family proteins in mice and did not affect the antitumor effect of DOX

**FIGURE 6:**
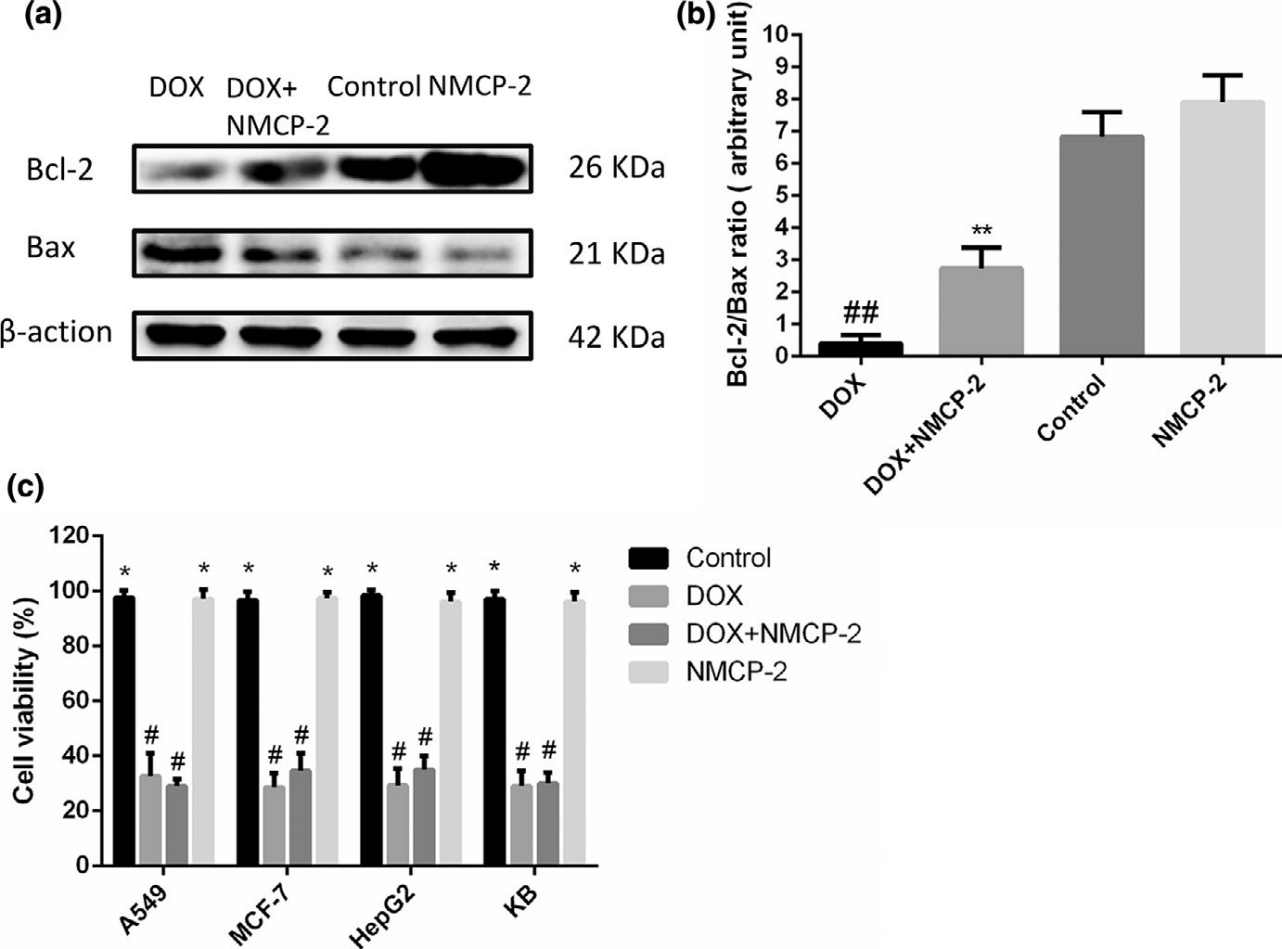
Effects of NMCP‐2 on expression patterns of Bcl‐2 and Bax in DOX‐induced mice, and the effect on the antitumor ability of DOX. (a) Protein levels of Bcl‐2 and Bax in myocardial tissues were determined using Western blotting. (b) Quantitative analysis of the ratio of Bcl‐2 to Bax in protein expression was evaluated. (c) A series of cancer cell lines, including A549, MCF‐7, HepG2, and KB cells, were treated with DOX (2 μM) in presence or absence with NMCP‐2 (15 μM for 4 h prior to DOX exposure) for 24 h. Cell viability was determined using MTT assay. Results are represented as means ± *SD* from three independent experiments. ##*p* < .01 and #*p* < .05 versus Control; **p < .01 and *p < .05 versus DOX‐induced cells

Western blotting was used to further evaluate the effect of NMCP‐2 on the expression of Bax and Bcl‐2 in myocardial tissues. As shown in Figure 6a, compared with the control group, the expression of Bax in the DOX‐induced group was significantly up‐regulated, and the expression of Bcl‐2 was significantly down‐regulated. However, pretreatment with NMCP‐2 reversed these phenomena. Moreover, Bcl‐2/Bax expression ratio showed that NMCP‐2 regulated the disordered expression of Bcl‐2 and Bax in DOX‐induced myocardial tissues (Figure 6b).

The effect of NMCP‐2 on the antitumor ability of DOX was finally evaluated. As shown in Figure 6c, DOX exerted a significant inhibitory effect on the growth of a series of cancer cell lines, including A549, MCF‐7, HepG2, and KB cells. However, co‐treatment with NMCP‐2 did not affect the inhibitory effect of DOX on the growth of these tumor cells. This result indicated that NMCP‐2 had no influence on the antitumor effect of DOX.

## 4 DISCUSSION

DOX belongs to the family of anthracycline drugs and has provided tremendous help in cancer treatment since 1960 (Jain, 2000). However, the clinical application of DOX is limited by dose‐dependent cardiotoxicity, which can cause irreversible myocardial damage in cancer patients (Swamy et al., 2012). Therefore, it is necessary to identify active natural compounds that can protect against DOX‐induced cardiotoxicity. In our previous study, NMCP‐2 is a polysaccharide purified from *Morchella conica* that modulates oxidative stress by regulating signaling pathways (Xu et al., 2018). This study suggested that NMCP‐2 could prevent the chronic cardiac toxicity caused by DOX and did not affect its anticancer activity. To the best of our knowledge, this is the first report to demonstrate that NMCP‐2 (a polysaccharide purified from *Morchella conica*) may be a potential nutritional food supplement that could be used to prevent myocardial damage in cancer patients treated with DOX.

In the in vitro experiments of this study, pretreatment with NMCP‐2 can effectively improve cell viability and inhibit DOX‐induced LDH release in H9C2 cardiomyocytes, which is considered to be a biomarker of cardiotoxicity (Tikoo et al., 2011). Then in a chronic DOX mouse heart injury model, compared with the DOX‐induced myocardial injury model in mice, NMCP‐2 pretreatment decreased the heart index, and the activities of serum markers (CK‐MB, AST, and ALT), and improved the tissue damage of the heart. In addition, heart sections from DOX‐treated mice showed fragmentation and degeneration of the myocardial tissue, manifested by focal myofibrillar loss and increased cardiac fibrosis, which were significantly improved by NMCP‐2 pretreatment. These results indicated that NMCP‐2 plays an important protective role against DOX‐induced myocardial damage in vivo and in vitro.

Numerous studies have also demonstrated that the underlying mechanism of DOX‐induced cardiotoxicity is closely related to oxidative stress in cardiomyocytes (Songbo et al., 2019; Yao et al.). In this process, some fixed antioxidant enzymes appear in the cells to reduce ROS levels and improve cell survival (Yu, Kang, et al., 2020). These antioxidant enzymes mainly include GSH, CAT, and SOD. ROS production attacks polyunsaturated fatty acids in biological membranes, triggers lipid peroxidation, and forms lipid peroxides such as MDA. SOD activity indirectly reflects the ability to scavenge free radicals, while the MDA level reflects the severity of ROS attacks (Sugden & Clerk, 2006). In this experiment, the results showed that NMCP‐2 inhibited DOX‐induced myocardial oxidative damage, as evidenced by the up‐regulation of SOD, CAT, and GSH levels in mice. In addition, NMCP‐2 significantly reduced the content of MDA, the final product of lipid hydrogen peroxide. Therefore, these results indicated that the cardioprotective effect of NMCP‐2 on DOX‐induced cardiotoxicity may be attributed to reduced oxidative stress.

Previous studies have shown that apoptotic death of cardiomyocytes is the most direct cause of DOX‐induced cardiotoxicity (Kalay et al., 2006; Pan et al., 2021). Apoptosis depends on the activation of caspase, and cleaved caspase‐3 is the key executor of apoptosis (Kalyanaraman et al., 2002). In this study, the protective effect of NMCP‐2 on apoptosis was confirmed by detecting caspase‐3 expression in the heart by immunohistochemistry.

There are two different pathways to stimulate caspase: the intrinsic (mitochondria‐dependent) apoptosis pathway and the extrinsic (death‐receptor‐dependent) pathway (Pryor et al., 2006), and the mitochondria‐dependent apoptotic pathway is the main mechanism underlying DOX‐induced cardiomyocyte apoptosis (Shi et al., 2018; Xiao et al., 2012; Zhuang et al., 2021). In the process of DOX metabolism, ROS generated in the mitochondria cause the loss of mitochondrial membrane potential, which leads to the release of cytochrome C. The released cytochrome C finally activates caspase‐3 and initiates the apoptotic degradation (Elmore, 2007). Our research results showed that NMCP‐2 can significantly prevent DOX‐induced cytochrome C release, caspase‐3 activation, and structural damages of mitochondria in vivo. Previous studies have been suggested that the activation of the mitochondrial‐dependent apoptosis pathway depends on p53 tumor suppressor protein, which is also related to the apoptosis of cardiomyocytes induced by DOX (Yoshida et al., 2009). P53 is a transcription factor that directly regulates the gene products of Bcl‐2 family proteins (including Bax pro‐apoptotic proteins and Bcl‐2 anti‐apoptotic proteins). Bcl‐2 prevents the release of apoptosis‐forming factors, such as cytochrome C, from the mitochondria and exerts anti‐apoptotic effects. Bax interacts with the voltage‐dependent ion channels on the mitochondria to mediate the release of cytochrome C. Therefore, p53 up‐regulates the expression level of Bax and down‐regulates the expression of Bcl‐2 to complete the promotion of apoptosis (Cory & Adams, 2002; Sun et al., 2013). Our results showed that the expression of p53 in the DOX‐induced group increased significantly, but the expression of p53 was almost reduced to the level of the control group after NMCP‐2 pretreatment. Compared with the DOX‐induced group, Bax protein expression increased and Bcl‐2 protein expression increased after NMCP‐2 pretreatment. Thus, NMCP‐2 can up‐regulate Bcl‐2 expression and down‐regulate Bax expression in myocardial tissues.

Another issue discussed in this study is the effect of NMCP‐2 on the antitumor activity of DOX. We doubt whether the addition of NMCP‐2 will change the anticancer function of DOX. Therefore, a series of cancer cell lines (A549, McF‐7, HepG2, and KB cells) were studied in vitro. Our results showed that compared with DOX alone, treatment with NMCP‐2 and DOX did not decrease the apoptosis of the cancer cells. These results indicated that NMCP‐2 did not affect the anticancer effect of DOX.

## 5 CONCLUSIONS

In summary, NMCP‐2 exerts a definite effect on DOX‐induced myocardial injury. This effect may be associated with the inhibition of oxidative stress and subsequent mitochondria‐dependent apoptotic pathways. Therefore, NMCP‐2 as a natural bioactive compound can be a potential food supplement for reducing the cardiotoxicity caused by DOX.

## CONFLICT OF INTEREST

The authors confirm that they have no conflicts of interest with respect to the work described in this manuscript.

## AUTHOR CONTRIBUTIONS

**Na Xu**: Conceptualization (supporting); formal analysis (equal); investigation (equal); methodology (supporting); visualization (equal); writing‐original draft (lead); writing‐review & editing (lead). **Yi Lu**: Conceptualization (lead); formal analysis (equal); data curation (equal); investigation (equal); methodology (lead); writing‐original draft (supporting); writing‐review & editing (supporting). **Xinmiao Yao**: Investigation (equal); methodology (equal); resources (equal); writing‐original draft (equal); writing‐review & editing (equal). **Rui Zhao**: Formal analysis (equal); investigation (equal); methodology (equal); writing‐original draft (equal). **Zhebin Li**: Formal analysis (equal); investigation (equal); methodology (equal); writing‐original draft (equal). **Jialei Li**: Formal analysis (equal); investigation (equal); methodology (equal); writing‐original draft (equal). **Yinglei Zhang**: Formal analysis (equal); investigation (equal); methodology (equal); writing‐original draft (equal). **Bo Li**: Formal analysis (equal); investigation (equal); methodology (equal); writing‐original draft (equal). **Ye Zhou**: Formal analysis (equal); investigation (equal); methodology (equal); writing‐original draft (equal). **Huifang Shen**: Formal analysis (equal); investigation (equal); methodology (equal); writing‐original draft (equal). **Liqun Wang**: Investigation (equal); methodology (equal); writing‐original draft (equal). **Kaixin Chen**: Methodology (equal); writing‐original draft (equal). **Li Yang**: Conceptualization (equal); funding acquisition (equal); investigation (equal); project administration (equal); supervision (equal); writing‐review & editing (equal). **Shuwen Lu**: Conceptualization (equal); funding acquisition (equal); investigation (equal); project administration (equal); supervision (equal); writing‐review & editing (equal).

## ETHICAL APPROVAL

Mice were complied with the guidance of Institutional Animal Care and Use Committee of Jilin University under the approved protocol number KT202004023.

## Data Availability

All the data used in this study can be made available upon reasonable request.
